# Krukenberg tumor arising from gastric cancer presenting as Pseudo-Meigs' syndrome: a case report and literature review

**DOI:** 10.3389/fmed.2024.1427568

**Published:** 2024-09-13

**Authors:** Shikang Qiu, Huihui Jiang, Hanxiao Ding, Limin Feng

**Affiliations:** ^1^Department of Gynecology, Beijing Tiantan Hospital, Capital Medical University, Beijing, China; ^2^Department of Clinical Laboratory, Qingdao Central Hospital, University of Health and Rehabilitation Sciences (Qingdao Cancer Hospital), Qingdao, Shandong, China

**Keywords:** Krukenberg tumor, Pseudo-Meigs' syndrome, ovary, dyspnoea, gastric cancer

## Abstract

Krukenberg tumor is a relatively uncommon metastatic ovarian cancer, typically presenting with abdominal pain and distension, primarily due to bilateral ovarian involvement. Pseudo-Meigs' syndrome, caused by a Krukenberg tumor originating from gastric cancer, is extremely rare. In this study, we report the case of a 39-year-old woman who presented with unusual manifestations of a Krukenberg tumor, where abdominal distension and dyspnea were the primary symptoms. After surgical treatment, a histopathological examination of the ovary revealed the presence of signet ring cell carcinoma. We concluded that this case coincided with Pseudo-Meigs' syndrome. Clinicians should note that Pseudo-Meigs' syndrome should be considered in patients with Krukenberg tumor, ascites, and pleural effusion, as resection of the tumor may provide long-term palliation.

## 1 Introduction

Meigs' syndrome refers to the presence of benign ovarian tumors accompanied by pleural effusion and ascites. Pseudo-Meigs' syndrome, on the other hand, is a clinical manifestation where malignant ovarian tumors are associated with similar benign ascites and pleural effusion. Krukenberg tumor was first reported by the German gynecologic pathologist Friedrich Ernst Krukenberg in 1896 (2). It is a rare metastatic signet ring cell carcinoma of the ovary, accounting for ~1% of all ovarian tumors (3). In most cases, the stomach is the primary site of Krukenberg tumor (5). Abdominal mass, abdominal pain, and abdominal distension are common symptoms in patients with Krukenberg tumor (6). However, symptoms of the primary tumor are often subtle, and the lack of distinctive phenotype in tests can lead to missed diagnosis or misdiagnosis.

In this report, we present a case of gastric cancer with Krukenberg tumor manifesting as Pseudo-Meigs' syndrome, with dyspnea being the first symptom. We also review similar cases to better understand the clinical features of such presentations. This case report also highlights the importance of maintaining a broad diagnostic perspective when evaluating patients with symptoms that may suggest a specific pathological process to avoid missed diagnosis or misdiagnosis.

## 2 Case description

**Figure 1:**
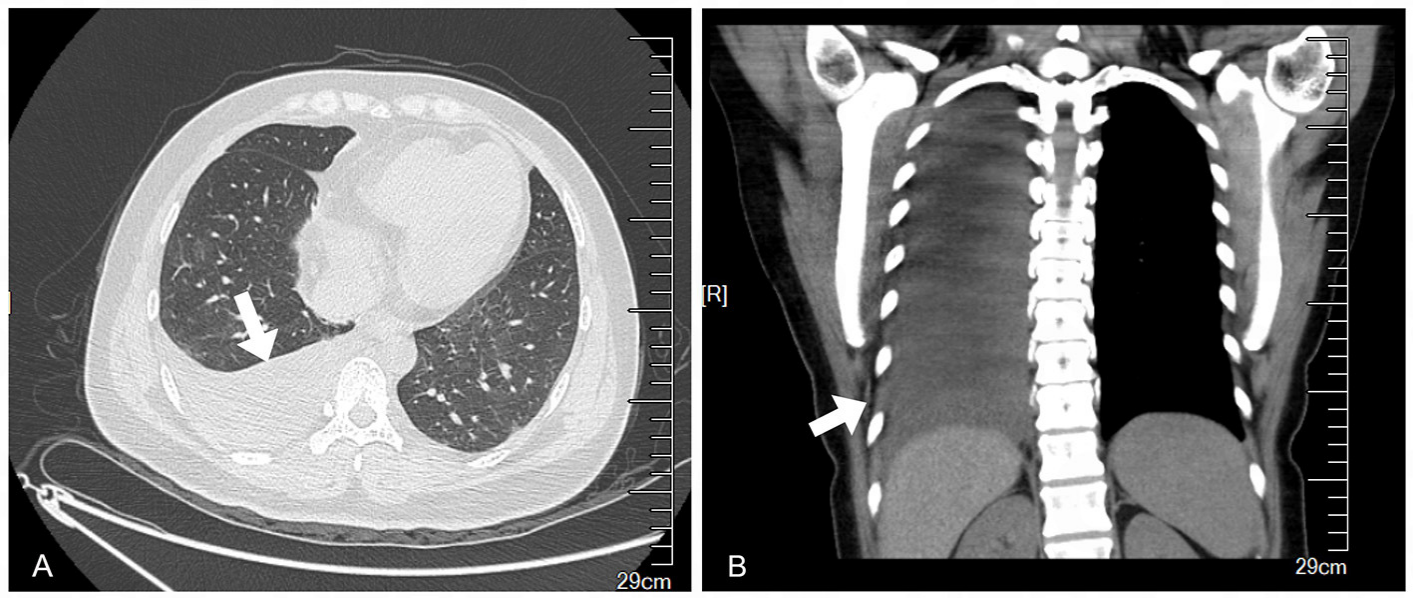
Preoperative chest CT showed massive pleural effusion on the right side and partial dilatation of the lower lobe. **(A)** Transverse plane. **(B)** Coronal plane.

A 39-year-old female patient presented with symptoms of cough and fever that persisted for 2 months before treatment. Over time, she developed chest pain, chest tightness, and shortness of breath. She initially went to the respiratory department, and a computed tomography (CT) scan revealed pleural effusion (Figure 1). However, anti-infective treatment with cefaclor proved ineffective.

**Figure 2:**
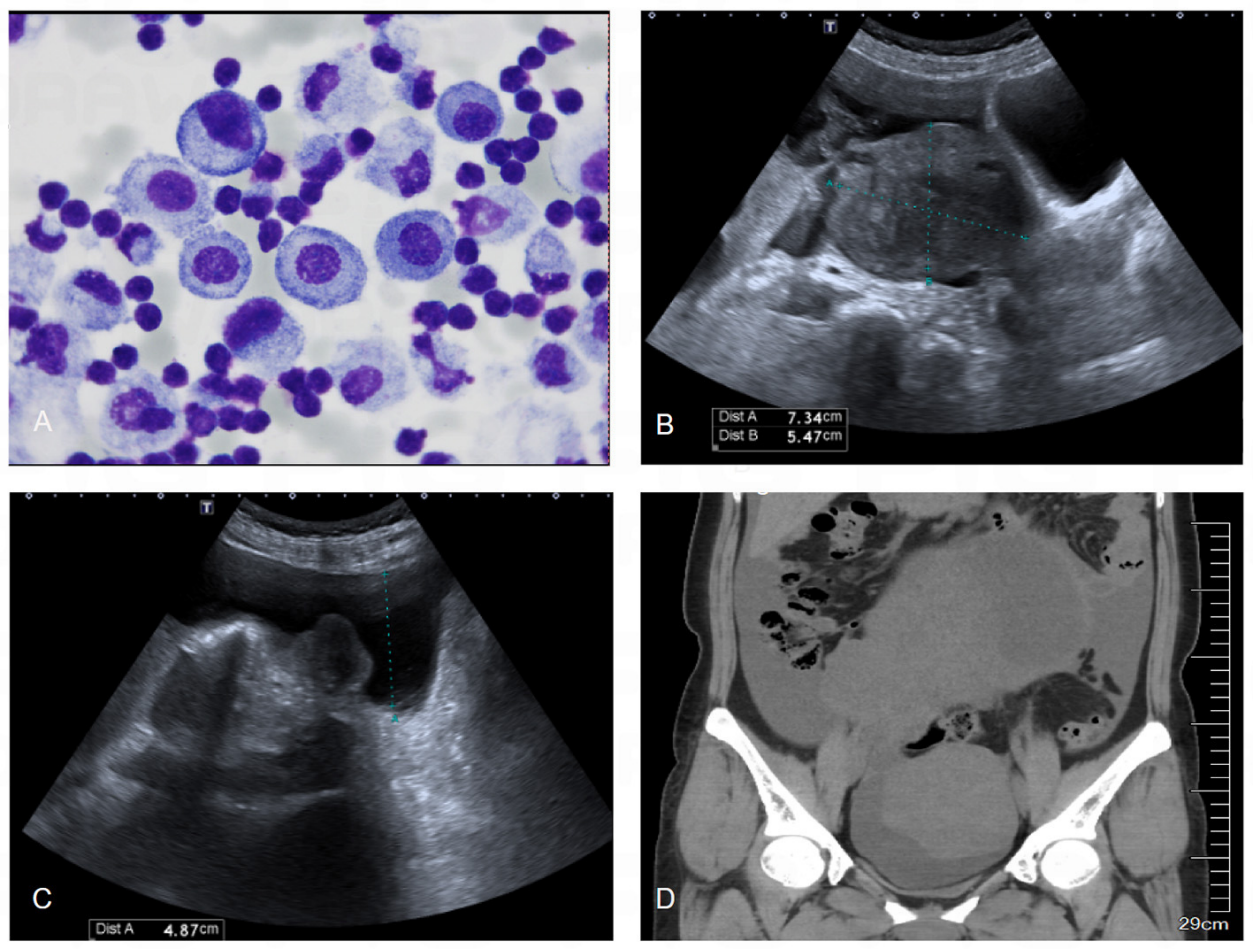
Results of abdominal ultrasound, pelvic CT and pleural effusion cytology. **(A)** Cytological examination of pleural effusion showed that the cells were mainly lymphocytes, and no malignant cells were found. **(B)** Abdominal ultrasound showed a heterogeneous echogenic mass about 16.5 × 15.2 × 10.7 cm in size in the pelvis, which was isoechoic, mainly solid, regular in shape, and clear in boundary. **(C)** Abdominal ultrasound showed a large number of fluid dark areas in the pelvic cavity, which were deep in the pelvic cavity, about 1.8 cm. **(D)** CT of the pelvic cavity showed a large cystic and solid mass in the pelvis, with a larger cross-sectional area of about 190 × 116 mm.

Subsequently, the clinician performed thoracocentesis and drained approximately 450 ml of pleural fluid. Analysis of the pleural effusion revealed an exudate mainly composed of mononuclear cells, with no evidence of tumor cells (Figure 2A). Her serum tumor marker levels were as follows: Alpha-Fetoprotein (AFP) 6.26 ng/ml, Carcinoembryonic Antigen (CEA) 1.46 ng/ml, Cancer Antigen 125 (CA-125) 270.9U/ml, Carbohydrate Antigen 19-9 (CA19-9) 38.56U/ml, Squamous Cell Carcinoma Antigen (SCC) 1.40 ng/ml, Human Epididymis Protein 4 (HE4) 52.67 pmol/L, and a Roma index of 9.8%.

An ultrasound examination revealed a cystic-solid mass (17.3^*^19.4^*^9.7 cm) in the right adnexal region, which was suspected to originate from the right ovary (Figures 2B, C). Further pelvic CT imaging revealed a large mass in the pelvic and abdominal cavities, with a large amount of effusion in both the pelvic and abdominal cavities as well as the right pleural cavity (Figure 2D). Given these findings, the clinician suspected Pseudo-Meigs syndrome and referred her to the gynecology department for surgery.

During laparotomy, ~2,000 ml of clear brown-yellow ascites were found in the abdominal cavity. Scattered miliary nodules were observed on the peritoneum and intestinal canal, along with a 20^*^18^*^10 cm cystic-solid mass in the right ovary. An intraoperative pathology report showed a poorly differentiated carcinoma in the right adnexa, with a high probability of signet cell carcinoma.

The patient underwent radical lymphadenectomy, total hysterectomy, and bilateral salpingo oophorectomy. Postoperative pathological examination confirmed the presence of signet ring cells filled with mucin in the right ovarian stroma, which was characteristic of a Krukenberg tumor. The results of immunohistochemistry and special staining were as follows: vimentin (interstitial +), CD10 (partial parenchymal cell +), a-inhibin (partial stromal cell +), Her-2 (1+, positive control 3 +), CK7 (+), CK20 (+), CK (+), CDX-2 (+), Villin (+), CLDN18.2 (+), SATB2 (-), CA-125(-), AB-PAS (PAS+), and reticulation (+). These findings suggest that the Krukenberg tumor involving the ovary originated from the gastrointestinal tract. Ascites examination showed scattered atypical cells, possibly tumor cells, with PAS (+) suggesting a likely origin from the digestive system.

She was diagnosed with poorly differentiated adenocarcinoma of the cardia and gastric angles, with the majority of the tumor composed of signet ring cell carcinoma, based on gastroscopy and pathology examinations conducted after surgery. This confirmed the diagnosis of Pseudo-Meigs' syndrome caused by a Krukenberg tumor. After the surgery, the pleural effusion resolved, her respiratory symptoms improved, and the ascites significantly decreased. She was then transferred to an oncology hospital for chemotherapy.

## 3 Discussion

Many clinicians broadly classify primary metastatic ovarian tumors originating from the gastrointestinal tract as Krukenberg tumors (10). However, the diagnosis of Krukenberg tumor is based on the WHO diagnostic criteria, which were established from the pathological descriptions by Novak et al.

These criteria include the presence of ovarian stromal infiltration, mucin-producing signet ring cells, and sarcomatous hyperplasia of the ovarian stromal tissue. The presence of signet ring cells is the key diagnostic feature of Krukenberg tumor (11). Krukenberg tumor is a selectively metastatic tumor, most commonly forming along the gastric-ovarian metastatic axis.

Interestingly, gastric cancer can metastasize selectively to the ovary without involving other tissues. However, the exact pathway and mechanism of gastrointestinal tumor metastasis to the ovary remain unclear. At present, three possible metastatic pathways exist: lymphatic metastasis, hematogenous (blood) metastasis, and transcoelomic (abdominal implant) metastasis (12).

Due to the low incidence of Krukenberg tumors, they are often misdiagnosed as primary gynecological tumors. Compared with primary ovarian cancer, the clinical manifestations of Krukenberg tumor are less specific, with common symptoms including abdominal pain and distension, often accompanied by ascites. These symptoms are followed by clinical symptoms related to the primary tumor, such as nausea and hematemesis (6, 13).

However, in our case, the patient initially presented with dyspnea and symptoms resembling a respiratory infection, leading her to first seek treatment in the respiratory department, where she was treated for inflammation. It was only later, after a pelvic mass was identified, that she was referred to the gynecology department. She had obvious respiratory symptoms and a large amount of pleural effusion, resulting in a few weeks of hospitalization before the gynecologist considered that the patient's symptoms might be consistent with Meigs' syndrome or Pseudo-Meigs' syndrome.

Preoperative ultrasound showed a solid-cystic mixed ovarian mass with malignant characteristics that differed from the typical presentation of Meigs' syndrome, usually caused by benign ovarian tumors. This finding made us more inclined to diagnose Pseudo-Meigs' syndrome before surgery, although the primary site of the tumor in the digestive tract had not been confirmed at that time.

As is well known, Meigs' syndrome is rare and is characterized by benign ovarian tumors, including fibroma, theca cell tumor, Brenner tumor, and granulosa cell tumor, accompanied by pleural and peritoneal effusion, with fibroma being the most common type. A key feature of Meigs' syndrome is that ascites and pleural effusion typically resolve following tumor resection (14). In contrast, Pseudo-Meigs' syndrome presents similarly but involves different types of tumors, including primary malignant tumors, metastases, or other benign ovarian tumors (15). The specific nature of the pelvic mass can only be confirmed through surgical resection and histological examination.

In the past, there have been a few reported cases of Pseudo-Meigs' syndrome resulting from secondary ovarian tumors associated with gastrointestinal cancer, but Pseudo-Meigs' syndrome caused by Krukenberg tumors is extremely rare. In 1950, Dick et al. reported the first case of Pseudo-Meigs' syndrome resulting from a Krukenberg tumor of gastric cancer (1). However, only a limited number of cases have been reported since then. To the best of our knowledge, this case is the first report of Pseudo-Meigs' syndrome caused by a Krukenberg tumor of gastric cancer in China. Table 1 summarizes the reported cases of this disease, including our case and those reported locally in Spain and Japan.

**Table 1:** Pseudo-Meigs' syndrome caused by Krukenberg tumor of gastric cancer.

**References**	**Age**	**Chief complaint**	**Primary tumor**	**Histology**	**Onset**	**CA125 (U/ml)**	**Ascites (amount)**	**Pleural effusion (site)**	**Ovarian tumor**	**Coopho-rectomy**	**Follow-up**
Dick et al. (1)	50	Abdominal distension, dyspnoea	Stomach	ND	S	ND	Yes (800 ml)	Yes (left)	Bilateral	Yes	ND
Cetin et al. (4)	47	Cachexia, dyspnea after gastrectomy	Stomach	PDA	M	ND	Yes (large)	Yes (right)	Bilateral	Yes	Dead after 46 months
Bayod et al. (7)	51	Abdominal distension, dyspnoea	Stomach	PDA	M	217	Yes (small)	Yes (bilateral)	Bilateral	Yes	ND
Okazaki et al. (8)	54	Abdominal distension, cough	Stomach	PDA	S	215	Yes (200 ml)	Yes (bilateral)	Bilateral	Yes	Alive after 18 months
Horimatsu et al. (9)	50	Abdominal distension, dyspnoea	Stomach	PDA	S	489.7	Yes (large)	Yes (right)	Bilateral	Yes	Dead after 27 months
Present case	39	Abdominal distension, dyspnoea	Stomach	PDA	S	270.9	Yes (2000 ml)	Yes (right)	Unilateral	Yes	Alive

ND, not documented; PDA, poor-differentiated adenocarcinoma; M, metachronous; S, synchronous.

One notable feature across these cases is the younger age of onset in the patients. Krukenberg tumors are indeed more common in younger women than primary ovarian cancer, possibly due to the fact that functioning ovaries, with their abundant blood supply, are more prone to metastatic disease. Another key observation is that the primary site of the tumor in these patients is consistently the stomach, suggesting that gastric cancer may have a particular propensity to cause Pseudo-Meigs' syndrome when it metastasizes as a Krukenberg tumor (16).

Moreover, nearly all of the patients in these cases presented with symptoms of abdominal distension and dyspnea. Kiyokawa et al. studied 120 cases of Krukenberg tumor and found that abdominal swelling was the most common clinical manifestation, with only five patients reporting symptoms of dyspnea, chest pain, or cough (17). This finding indicates that dyspnea may be a more common symptom in cases of Pseudo-Meigs' syndrome caused by Krukenberg tumors.

In clinical practice, especially in respiratory departments, it is crucial not to overlook the possibility of abdominal pathology in patients presenting with dyspnea and pleural effusion. When symptoms such as abdominal pain, distension, or palpable lumps or effusions are present, further digestive and gynecological examinations should be conducted to improve the accuracy of the diagnosis.

In addition, in all the cases we reviewed, the primary tumors were consistently identified as poorly differentiated adenocarcinoma. The ovarian tumors in these cases were typically bilateral, and the onset of tumor metastasis was predominantly simultaneous, which are common characteristics of Krukenberg tumors (18). However, what sets Pseudo-Meigs' syndrome caused by a Krukenberg tumor of gastric cancer apart is that pleural effusion is more frequently observed on the right side, and most patients exhibit only mildly or moderately elevated levels of CA125.

Interestingly, in the reviewed cases of Pseudo-Meigs' syndrome caused by gastric cancer, including our case, CA-125 levels were elevated to < 500 U/ml, even in the presence of massive ascites, suggesting that CA-125 may not be a reliable marker for distinguishing between benign and malignant tumors and for assessing the extent of dissemination in such cases. In addition, it has been reported that Meigs' syndrome caused by benign tumors can lead to a significant increase in CA-125 levels (19, 20), reaching up to 1,835 U/ml. Therefore, when making a differential diagnosis, more attention should be paid to imaging findings and clinical symptoms, with pathological results serving as the definitive standard.

Finally, all of these patients underwent ovariectomy. Massive ascites and pleural effusion are characteristic manifestations of Pseudo-Meigs' syndrome, although the precise mechanism of their formation remains unclear. It is speculated that fluid from the tumor infiltrates the abdominal cavity through the serosa surface and then enters the pleural cavity, either unilaterally or bilaterally, via lymphatic vessels that connect the pleural cavity with the abdominal cavity (21). This process likely explains why ascites and pleural effusion typically resolve after the surgical removal of ovarian tumors.

However, in this particular case, while the pleural effusion quickly disappeared after surgery, the ascites persisted. The most likely explanation is that we found a large number of metastatic lesions in the peritoneum during the operation, contributing to both ascites associated with Pseudo-Meigs' syndrome and malignant ascites caused by these metastatic lesions. This persistence of ascites may indicate a poor prognosis for the patient.

In general, the detection of a Krukenberg tumor precedes the diagnosis of the primary tumor, which is often small, asymptomatic, and may not even be detected until several years after an ovariectomy, complicating the initial diagnosis. Since most primary lesions do not cause symptoms, patients are often unaware of the disease until ovarian metastasis has progressed.

Statistics show that only 20% to 30% of cases have a prior history of gastric cancer or other organ cancer (22). By the time the tumor is discovered, it is frequently at an advanced stage. Overall, the prognosis for Krukenberg tumor is poor, with a mean survival period of only 14 months (23). Differentiating between hydrothorax and ascites caused by tumors can be challenging, highlighting the importance of identifying the primary tumor that causes these symptoms before surgery to guide treatment as soon as possible.

There are currently no guidelines for the treatment of Pseudo-Meigs' syndrome caused by a Krukenberg tumor. At present, surgery is generally considered the main treatment approach. In addition to the removal of the primary tumor when feasible, both bilateral and unilateral ovarian metastases should be addressed simultaneously, along with the excision of the uterus, bilateral adnexa, and greater omentum.

Most of the cases we reviewed reported that patients experienced symptom relief, particularly with a reduction in hydrothorax and ascites, following surgical intervention. The survival time for patients who underwent surgery was significantly longer compared to those who only received a biopsy or did not undergo surgery at all. Therefore, for patients who are able to tolerate surgery, it is recommended to remove both the primary tumor and any metastases simultaneously. Even if the resection of the primary cancer is palliative, it can reduce the tumor burden, alleviate pain or obstructions, and ultimately improve the patient's quality of life.

In summary, we present a case of a Krukenberg tumor causing Pseudo-Meigs syndrome, where dyspnea was the first symptom. In this article, we aim to raise awareness of the possible clinical presentations of this tumor. When symptoms cannot be explained by routine examination, it is crucial to examine the patient thoroughly. Although Krukenberg tumor is a rare ovarian tumor, it should be considered in the differential diagnosis, particularly when patients, especially within the relevant age group, present with unusual symptoms such as dyspnea. Recognizing this syndrome can lead to a quicker diagnosis and appropriate treatment.

## Data Availability

The original contributions presented in the study are included in the article/supplementary material, further inquiries can be directed to the corresponding author.
